# Lumen-apposing metal stents (LAMS) versus plastic stents for EUS-guided drainage of walled-off necrosis (WON) (LVPWON): study protocol for a multicenter randomized controlled trial

**DOI:** 10.1186/s13063-018-2901-3

**Published:** 2018-10-11

**Authors:** Hui-Yun Zhu, Pei Xie, Ying-Xiao Song, Zhao-Shen Li, Zhen-Dong Jin, Yi-Qi Du

**Affiliations:** 1 Department of Gastroenterology, Changhai Hospital, Second Military Medical University/ Naval Medical University, 168 Changhai Road, Shanghai, 200433 China; 2 Digestive Endoscopy Center, Changhai Hospital, Second Military Medical University/ Naval Medical University, 168 Changhai Road, Shanghai, 200433 China; 3 Shanghai Institute of Pancreatic Diseases, Shanghai, China; 4 National Clinical Research Center of Digestive Diseases, Shanghai, China

**Keywords:** LAMS, DPPS, WON, Trial, EUS

## Abstract

**Background:**

Endoscopic ultrasonography (EUS)-guided drainage has become the first-line therapy for late peri-pancreatic fluid collection (PFC). Double pigtail plastic stents (DPPS) and lumen-apposing metal stents (LAMS) are commonly used for PFC drainage. Recently, a multi-institutional consensus on PFC drainage has recommended that LAMS should be the standard care for patients with walled-off necrosis (WON). However, given the poor quality of evidence, we aim to perform a large-scale randomized controlled trial to determine whether LAMS is superior to DPPS for WON drainage.

**Methods/design:**

The study is an open-label, prospective, parallel-group, superiority, multicenter randomized controlled trial. Two hundred and fifty-six patients with WON who will attend 18 tertiary hospitals in China will be randomly allocated to the LAMS or DPPS group before the procedure. The primary endpoint is the clinical success at one month after drainage (reduction in the size of WON to < 2 cm). Secondary endpoints include technical success, operation time, recurrence, adverse events, and secondary interventions.

**Discussion:**

The LVPWON trial is designed to determine whether LAMS is effective, safe, and superior to DPPS for WON drainage.

**Trial registration:**

ClinicalTrials.gov, NCT03027895. Registered on 14 January 2017.

**Electronic supplementary material:**

The online version of this article (10.1186/s13063-018-2901-3) contains supplementary material, which is available to authorized users.

## Background

Walled-off necrosis (WON) is a type of pancreatic fluid collection (PFC) that develops in the setting of acute or chronic pancreatitis, trauma, or pancreatic duct obstruction [1]. WON, a delayed complication of necrotizing pancreatitis usually occurring > 4 weeks following the onset of pancreatitis, is PFC surrounded by a radiologically identifiable capsule containing both solid and liquid components [1]. Most WONs are asymptomatic and resolve spontaneously. However, drainage is necessary when it becomes symptomatic or infected, or increases in size over the course of the illness.

In the past decade, the treatment of symptomatic WON has evolved from surgical to endoscopic necrosectomy. Endoscopic treatment has a reduced proinflammatory response compared to surgery for the treatment of WONs [2]. It is associated with lower rates of pancreatic fistula formation and shorter hospital stays [3, 4]. Endoscopic ultrasonography (EUS)-guided drainage has high technical and clinical success and is associated with low adverse events (AEs) making it the optimal drainage approach for WONs [5, 6].

Double pigtail plastic stents (DPPS) is the standard choice for pancreatic pseudocyst drainage with > 90% technical and clinical success rates [5]. DPPS has also been used for WON drainage [7]. Recently, a novel lumen-apposing metal stent (LAMS) with a larger luminal diameter (≥ 10 mm) has been successfully used for EUS-guided drainage of PFC [8]. A multi-institutional consensus made by 22 expert endosonographers recommended that LAMS should be the standard of care for WON drainage [9].

However, the safety of LAMS is still controversial. Some studies have reported that LAMS is superior to DPPS in terms of overall treatment efficacy (90% vs 81%) and a significantly lower number of procedures (2.2 vs 3.6) [10]. LAMS includes single-step deployment and has an anti-migration structure [11, 12]. Other studies have proposed that LAMS was associated with a significantly higher rate of bleeding compared with DPPS [13–15]. Moreover, high quality of evidence with regards to the efficacy and safety of LAMS for WON drainage in Chinese patients is lacking. Given the recent widespread use of LAMS in the management of WON, we designed this open-label, prospective, parallel-group, superiority, multi-center randomized controlled trial (RCT) to investigate whether LAMS is superior to DPPS for WON drainage.

## Methods/design

### Design

The LVPWON trial is an open-label, prospective, parallel-group, superiority, multi-center RCT designed to determine whether LAMS is effective and safe in the EUS-guided drainage of WON and superior to DPPS. Patients with WON admitted at 18 tertiary hospitals in China will be randomly allocated to the LAMS or DPPS group before the procedure. The study protocol has been approved by the Institutional Review Board and Ethics Committee of Shanghai Changhai Hospital. Recommendations for Interventional Trial (SPIRIT) Checklist have been provided (see Additional file 1).

#### Study population

**Fig. 1:**
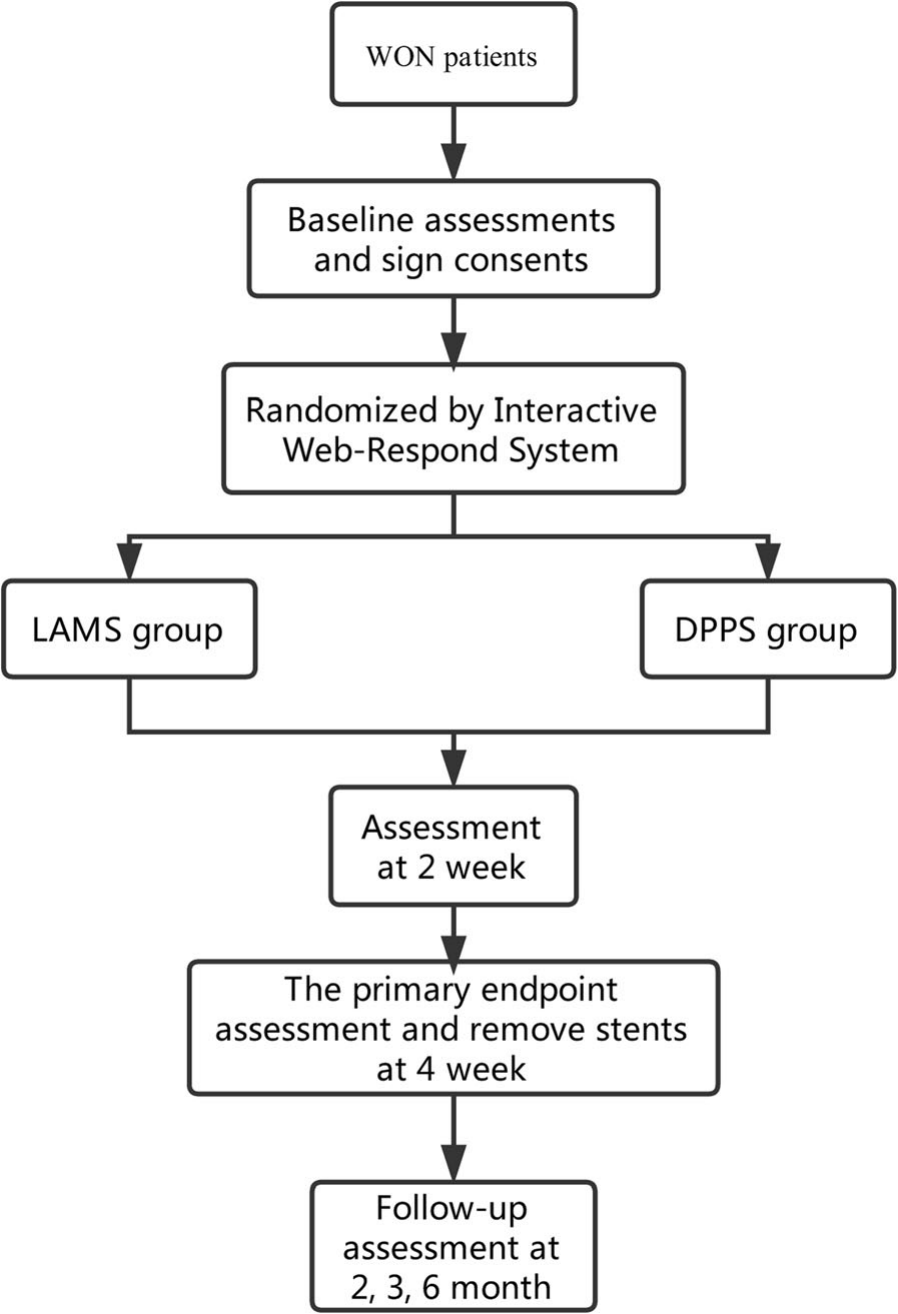
Research *flow chart*

This prospective study will be performed at the National Clinical Research Center for Digestive Diseases and 18 tertiary hospitals in China. All adult patients admitted with WON will be assessed for eligibility during their hospital admission. If patients are found to have solid debris in the PFC and fulfill all inclusion and exclusion criteria, they will be randomized (at a 1:1 ratio) to the LAMS or DPPS group after obtaining signed informed consent (Fig. 1). In this experiment, randomization will be performed using the Interactive Web-Respond System (IWRS).

The inclusion and exclusion criteria are listed in Table 1. All patients (aged ≥ 18 years) with WON requiring drainage and fulfilling the inclusion criteria will be eligible for enrollment. Investigators will inform the patients about the trial procedure. Patients will independently choose to participate in the trial and sign the informed consent. Any participant has the right to opt out of this trial at any time. Contraindications for EUS-guided drainage of WON will be determined by endoscopists or anesthesiologists before the procedure. CT images obtained from the 18 hospitals will be uniformly read by Changhai Hospital.

**Table 1:** Inclusion and exclusion criteria

Inclusion criteria	
Aged 18–80 years	
Individual with WON confirmed by CT and MRCP	
The size of WON ≥ 6 cm and located adjacent to the gastric wall	
Participants has symptoms related to the WON	
Written informed consent obtained	
Exclusion criteria	
Aged < 18 years or > 80 years	
Individual cannot accept the endoscopic procedure	
The distance between the stomach and the wall of the WON ≥ 1 cm	
Participant has blood coagulation dysfunction (platelet count < 50 × 109/L or INR > 1.5)	
Allergic to nickel titanium	
Suffering from severe lung or heart disease	
Pregnant and lactating women and those who are about to become pregnant soon	
Any other factors that are not suitable for inclusion or influence the individual’s participation in the study judged by researchers	

*WON* walled-off necrosis, *INR* international normalized ratio

#### Treatment protocol

**Fig. 2:**
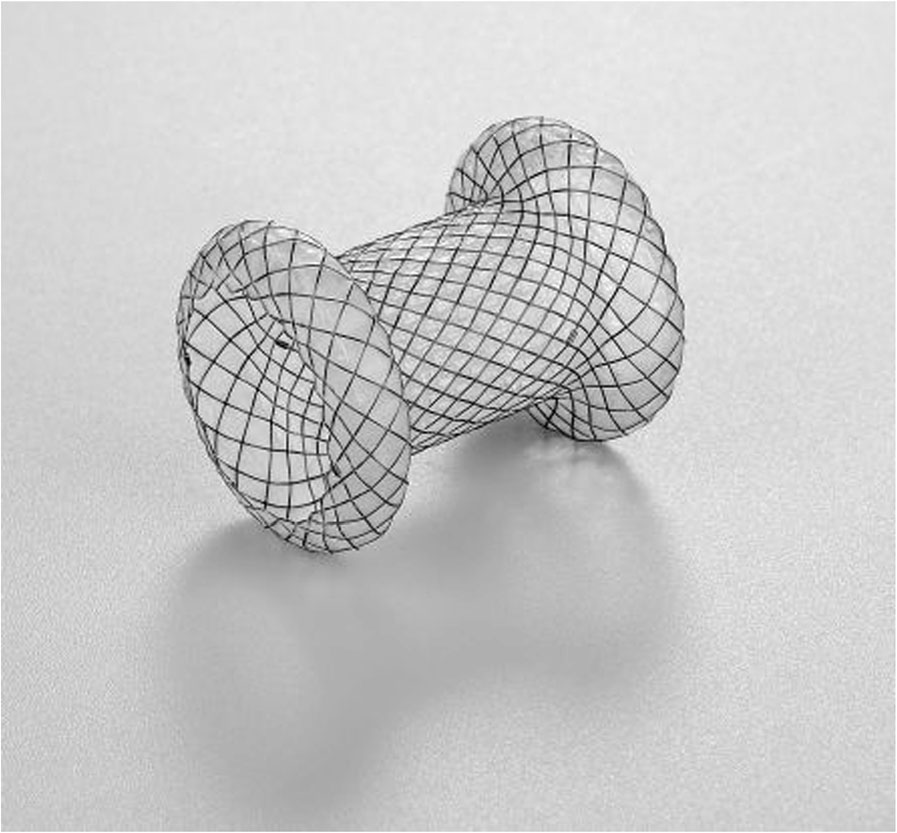
Lumen-apposing metal stent (Micro-TechCo. Ltd., Nanjing, China)

Before the procedure, if an individual meets the inclusion and exclusion criteria, the investigator will report this to the leader unit (Shanghai Changhai Hospital) and the participant will be randomized to receive either LAMS (Fig. 2) or DPPS for WON drainage. Randomization will occur in a 1:1 fashion with a random number table generated by IWRS, making it possible for all patients from different hospitals to be randomly assigned to each group.

The operation will be performed under mild sedation, monitored under anesthesia care or under general anesthesia, and prophylactic antibiotics will be used when appropriate. EUS-guided drainage will be performed by expert endosonographers after unified standard operation training. First, the position and size of the WON will be determined by endoscopic ultrasonography and the appropriate puncture point will be selected. Next, a 19G puncture needle will be inserted into the WON via the endoscope. Under fluoroscopy guidance, a 0.035 in. (1 in. = 0.025 m) yellow zebra guide wire will be inserted and coiled into the WON cavity. A Wilson COOK cystotome electric knife will be placed along the guide wire to cut the wall of stomach and the wall of the WON. Balloon expansion will be used if necessary. Subsequently, after drainage of the liquid content of the WON in to the stomach, LAMS or DPPSs would be placed to maintain the opening.

#### Data collection and follow-up

**Fig. 3:**
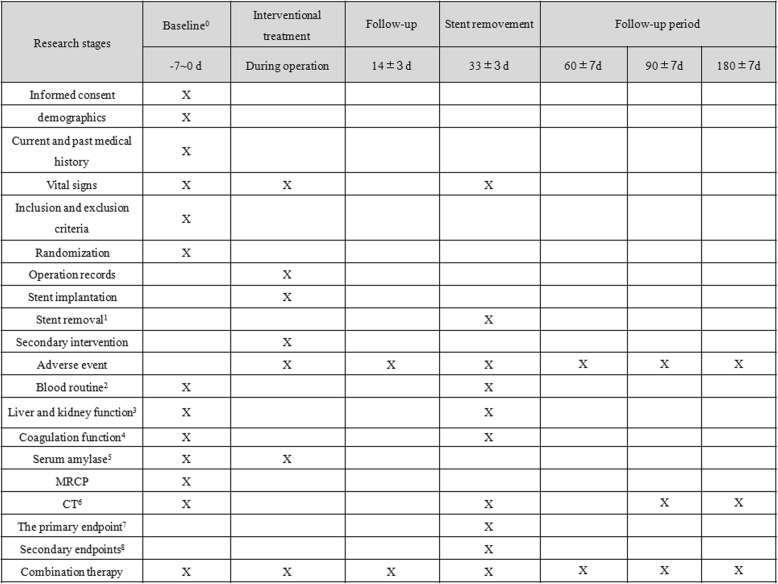
Schedule of enrollment, follow-up, and assessments. Basline^0^: screening of laboratory tests can be accepted the previous week in our hospital inspection report. Stent removal^1^: time from stenting to postoperative withdrawal (33 ± 3 days). Blood routine^2^: hemoglobin (HGB), erythrocyte count (RBC), platelet count (PLT), leukocyte count (WBC), neutrophil percentage (%), lymphocyte percentage (%). Liver and kidney function^3^: alanine aminotransferase (ALT), aspartate aminotransferase (AST), total bilirubin, direct bilirubin (DBIL), indirect bilirubin (IBIL), alkaline phosphatase (ALP), glutamyl transferase (GGT), blood urea nitrogen (BUN), creatinine (Cr). Coagulation function^4^: prothrombin time (PT), fibrinogen (FIB), activated partial thromboplastin time (APTT), thrombin time (TT). Serum amylase^5^: assessed 3 h and 24 h after the operation. CT^6^: preoperative and postoperative enhanced CT examination, 3 months and 6 months postoperative CT examination. The primary endpoint^7^: immediate success rate of surgery, drainage success rate 1 month after surgery. Secondary endpoints^8^: the incidence of clinical complications, operation time, the recurrence rate of pancreatic pseudocyst within 1 month and 1 month after the operation, and secondary interventions

Investigators will save a recording of the operation and note the procedure time. After EUS-guided drainage, patients will be sent back to the ward. Patients will be monitored for 3 h and 24 h serum amylase after the operation and prophylactic antibiotics will be administered postoperatively. Detailed data information can be found in the SPIRIT figure (Fig. 3). After the LAMS is implanted, if the WON needs to be cleaned, direct endoscopic necrosectomy (DEN) will be performed. Stents will be removed one month after implantation. If the clinical success criterion is not met at the one-month follow-up, the patient will be provided with further treatment that will be decided based on their individual needs. All individuals who participated in the trial will be followed up at one, three, and six months post operation, with a final follow-up to occur 12 months after the end of the trial for CT evaluation. The above data will be collected into case report forms (CRFs). Stent-related AEs and management will also be recorded and reported in the CRFs. Finally, CRFs will be summarized by the National Clinical Research Center for Digestive Diseases.

#### Outcomes

The primary outcome is the clinical success rate at one month after drainage. Clinical success is defined as reduction in the size of WON to < 2 cm. Within one month of the drainage, if the patient needs to change the treatment method (such as the additional placement of nasal cyst drainage tube; or the DPPS removed and replaced with a LAMS), it will be recorded as drainage failure. The secondary outcome is technical success, operation time, recurrence, AEs, and secondary interventions. The definitions of the primary and secondary outcomes are presented in Table 2.

**Table 2:** Definitions of the primary and secondary outcomes

Outcomes	Description
Clinical success	The diameter of the WON ≤ 2 cm
Technical success	LAMS or DPPS deployed successfully
Operation time	Time duration from the beginning to the end of the EUS-guided stent implantation procedure
Recurrence	After successful drainage, cyst reappears on imaging examinations with symptoms requiring intervention
Adverse event	
Perforation	Imaging manifests as pneumoperitoneum with peritoneal irritation syndrome
Bleeding	Any bleeding that requires intervention, blood transfusion, and hospital observation
Suprainfection	Postoperative fever, increased inflammatory index (CRP, PCT), or positive blood culture
Occlusion	The stent is filled with tissue or debris
Migration	The stent is completely or partially displaced into the WON or stomach
Others	Adverse events that occurred in the trial but not described above
Secondary intervention	Any endoscopic operation after stent is placed

*CRP* C-reactive protein, *PCT* procalcitonin

#### Statistical considerations

##### Sample size

The sample size calculation is based on a literature review and conference discussion regarding the clinical success rate of LAMS or DPPS drainage for WON. The LVPWON trial is a superiority trial in which the sample size calculation was based on the assumption that the incidence of the primary endpoint with LAMS and DPPS is 90% and 75%, respectively. Assuming a one-side alpha (type I error) of 0.025 and a power of 85% (beta, a type II error, was taken as 0.15), we used the following formula to calculate sample size and found that 256 patients (128 per condition) would be necessary, including a possible dropout rate of 10%.

$$ n={\frac{\mu_{1-\alpha}\sqrt{2\overline{p}\left(1-\overline{p}\right)}+{\mu}_{1-\beta}\sqrt{p_T\left(1-{p}_T\right)+{p}_C\left(1-{p}_C\right)}}{{\left({p}_T-{p}_C\right)}^2}}^2 $$

In this formula, *p*_*T*_ represents expected success rate of the LAMS group, *p*_*C*_ indicates expected success rate of the DPPS group, $\overline{p}$ is average success rate of the two groups, and *μ* represents the quantile of standard normal distribution.

##### Data management

Patient characteristics will be recorded in a CRF. An electronic data collection system (electronic data capture [EDC]) will be used to complete the trial data collection. The EDC system has been rigorously tested to fully meet the requirements of the “Quality Management Practice for Medical Device Clinical Trials” and “Technical Guidelines for Clinical Trial Data Management Work.” Before the system is officially launched, training tests will be conducted on relevant users to ensure that the system meets the trial requirements. After the official launch, related personnel will receive the account number and password. The account is bound to the user’s role and permissions: the user must properly maintain the account information, not inform others about the account information, and exercise appropriate rights for others. Clinical auditors will monitor the clinical trial center’s work at least once a month. This trial has an interim analysis once half of the required number of the patients have been enrolled, during which the data will be analyzed. If a serious AE is identified, the study will be terminated immediately.

##### Statistical analysis plan

Categorical data will be described using frequency and composition ratios. Continuous data will be described using mean, standard deviation, maximum, minimum, and median, as well as 25th and 75th quantiles. Baseline demographic analysis based on the descriptive analysis, the likelihood ratio χ2 test will be used for the comparison between the categorical data groups. Fisher’s exact probability method will be used when theoretical frequency of cells exceeding 25% is < 5. Normally distributed continuous datasets will be compared using a group *t*-test. For non-normally distributed continuous data, Wilcoxon rank sum test will be used for comparison between groups. This is a multi-center study. The test conditions of each center are not identical (such as operators, etc.) and the outcomes may be different. For the primary outcome, comparison between groups will be performed using the CMH (Cochran–Mantel–Haensel) χ2 analysis to adjust for the central effect. The differences between the success rates of the groups and their 95% confidence intervals (CI) will also be estimated. AEs will be described by the number and incidence of AEs; the likelihood ratios between groups will be compared using the χ2 test or Fisher’s exact probability test. Major investigators will have access to the final trial dataset and the statistician involved will be blinded to the treatment assignments. Statistical analysis will be performed with two-sided tests at a level of significance of 0.05 (except where specified). Data analysis will be conducted using SAS^®^9.4 statistical software.

## Discussion

The LVPWON trial has been designed to answer the question of whether LAMS is superior to DPPS for EUS-guided drainage of WON with regards to the clinical success rate and the incidence of AEs. We also want to prospectively investigate the risk factors for success of treatment and complications of EUS-guided drainage, which will provide a reference for the clinical treatment of WON.

EUS-guided drainage has been maturely applied in late PFC with well-defined inflammatory walls [7, 16–18]. Stents used for drainage are diverse. DPPS used in the management of PFC was first reported in the 1980s [19]. Subsequently, self-expanding metal stents (SEMS) and double-flanged LAMS have become the most popular drainage stents for PFC [8, 11, 12].

Due to their small diameter, several DPPSs are needed to achieve adequate drainage of a WON. Metal stents that have a larger diameter are theoretically considered superior to DPPS as they allow for the possibility of WON debridement. A previous study directly comparing DPPS and LAMS demonstrated that the latter had a success rate of 90%, which was higher than the former, and a significantly fewer number of procedures were required with LAMS for WON resolution [10]. In terms of AEs, stent occlusion seems more likely to occur in WON treated with DPPS [10]. Furthermore, new technology associated with LAMS enhance the drainage effect [20]. The procedure of LAMS deployment has become much easier and more economical.

However, with the increasing use of LAMS for WON drainage, LAMS-related bleeding has been more frequently reported [13–15]. In combined endoscopic and percutaneous drainage for symptomatic WON, LAMS did not reduce the time to percutaneous drain removal and was not associated with fewer AEs [21]. Furthermore, LAMS is substantially more expensive than DPPS.

Recently, a consensus guideline formulated by the Asian EUS group RAND/UCLA expert panel raised the issue of stent selection [5]. There are currently no randomized data addressing how LAMS compare with DPPS for WON drainage. An ongoing RCT observed serious LAMS-related AEs, including delayed bleeding in 50% of patients (6/12), buried stent syndrome, and biliary stricture [14]. However, our team had analyzed our center’s experience and determined that the use of LAMS is safe [22].

The LVPWON trial was initiated by Changhai Hospital, which is a National Clinical Research Center for Digestive Diseases. This is the largest prospective, open-label, parallel-group, superiority, multi-center RCT to address the appropriate selection of the stent for WON drainage. The trial includes 18 Chinese tertiary hospitals. Despite discrepancies in diagnostic and operational skill levels at different centers, as the research initiator, Changhai Hospital will provide technical training. Only experienced endosonographers will perform drainage procedures. The trial will also adopt “centralized readings” to reduce the heterogeneity between centers. Given the differences in the type of stent that will be placed, endoscopists and patients will not be blinded to the treatment allocation. However, the outcomes of the trial are unlikely to be affected by the patient’s psychological factors.

In conclusion, the major focus of the LVPWON trial is to prospectively compare the efficacy and safety of LAMS and DPPS in EUS-guided drainage of WON and to identify the risk factors associated with LAMS-related complications, thus further benefiting WON patients treated with LAMS.

### Trial status

This multicenter RCT is expected to begin enrolling patients on 30 September 2018. Protocol version number: V 2.0, 2017-10-10. The approximate date when recruitment will be completed is 30 September 2019.

## Additional file

Additional file 1: SPIRIT checklist. (DOC 118 kb)

## Abbreviations

CI: Confidence interval
CMH: Cochran–Mantel–Haensel
CRF: Case report form
CRP: C-reactive protein
DEN: Direct endoscopic necrosectomy
DPPS: Double pigtail plastic stents
EDC: Electronic data capture
EUS: Endoscopic ultrasonography
IWRS: Interactive Web-Respond System
LAMS: Lumen-apposing metal stents
LVPWON: LAMS vs DPPS trial
PCT: Procalcitonin
PFC: Pancreatic fluid collection
SEMS: Self-expanding metal stent
WON: Walled-off necrosis
